# In Situ Toxicity Reduction and Food Safety Assessment of Pak Choi (*Brassica campestris* L.) in Cadmium-Contaminated Soil by Jointly Using Alkaline Passivators and Organic Fertilizer

**DOI:** 10.3390/toxics11100824

**Published:** 2023-09-29

**Authors:** Wei Jiao, Zhi Li, Ruiping Li, Jiafeng Guo, Xiaoshu Hou, Xi Zhang, Fangli Wang

**Affiliations:** 1 Shandong Provincial Key Laboratory of Water and Soil Conservation and Environmental Protection, College of Resources and Environment, Linyi University, Linyi 276000, China; jiaowei19856261@163.com; 2 School of Resources and Environment, Qingdao Agricultural University, Qingdao 266109, China; 3 School of Geography and Tourism, Qufu Normal University Rizhao Campus, Rizhao 276800, China; 4 Qingdao Hairun Water Group Co., Ltd., Qingdao 266000, China; 5 Chinese Academy of Environmental Planning, Beijing 100012, China; 6 Institute of Agricultural Resources and Regional Planning, Chinese Academy of Agricultural Science, Beijing 100081, China

**Keywords:** cadmium (Cd) toxicity, organic fertilizer, alkaline passivator, soil remediation, food safety

## Abstract

An economical and effective method is still lacking for cadmium (Cd) toxicity reduction and food product safety improvement in soil–vegetable systems. Therefore, this study aimed to reduce the Cd toxicity to pak choi (*Brassica campestris* L.) by jointly using passivators and organic fertilizer, highlighting food products’ safety based on pot experiments. The results showed that compared with the control, organic fertilizer decreased the Cd content in edible parts and the soil’s available Cd by 48.4% and 20.9% on average, respectively, due to the 0.15-unit increases in soil pH. Once jointly applied with passivators, the decrements increased by 52.3–72.6% and 32.5–52.6% for the Cd content in edible parts and for the soil’s available Cd, respectively, while the pH increment increased by 0.15–0.46 units. Compared with the control, the transport factor of Cd was reduced by 61.9% and 50.9–55.0% when applying organic fertilizer alone and together with the passivators, respectively. The combination treatment of biochar and organic fertilizer performed the best in decreasing the Cd content in the edible parts and the soil’s available Cd. The combination treatment of fish bone meal and organic fertilizer induced the greatest increases in soil pH. The grey relational analysis results showed that the combination treatment of biochar and organic fertilizer performed the best in reducing the potential Cd pollution risk, thereby highlighting the vegetable food safety. This study provides a potential economical and effective technology for toxicity reduction and food safety in Cd-polluted soil.

## 1. Introduction

Global soil contamination with cadmium (Cd) is experiencing an alarming escalation due to the rapid growth of industrial activities [1,2]. Among all soil pollutants, Cd pollution stands out as the most severe, surpassing the allowed threshold by over 7% [3]. International regulatory bodies such as the Food and Agriculture Organization (FAO) and the World Health Organization (WHO) have established a safety limit of 1 mg kg^−1^ for Cd concentration in soil [4]. Cd is a major pollutant in soil worldwide, and Cd pollution in the soil environment is the most serious in China [5]. Cd can be enriched in the human body through the food chain and potentially damage human health [6]. The safety of agricultural food products contaminated by Cd has been a key issue in environmental science for decades. The methods to reduce Cd pollution in soil mainly include physical, chemical, biological, agroecological, and combined technologies [7]. As a typical chemical technology, passivation technology has been widely used in the remediation of Cd-polluted soil due to its advantages of low cost, simple operation, and less harm to soil [8]. Studies have shown that the cadmium removal rates of red mud at 35 °C and pH 9 are 97.31% and 96.20%, respectively [9]. Zhou et al. [10] found that high passivation rates of Cd, Cu, and Pb could be obtained by adding sawdust charcoal and wheat straw charcoal to composted pig manure. However, an economical and effective method is still lacking for reducing Cd toxicity and ensuring food products’ safety while increasing the food production in soil–vegetable systems.

Organic fertilizer (OF) is a popular fertilizer that highly increases agricultural production [11]. Organic fertilizer, which excludes the direct deposition of animal excreta by grazing animals, encompasses organic amendments such as animal manure, sewage sludge, compost, and other biowastes [12]. Previous studies have reported that OF easily combines with heavy metals to form steady-state compounds, reduce their bioavailability, and inhibit plant enrichment [13]. However, some studies have indicated that dissolved organic matter released by OF itself and the decomposition process chelates with Cd to improve its plant availability [14]. The effects of OF on the Cd availability in soil and the Cd accumulation in crops remain controversial [15]. To overcome the OF-induced problems in agricultural production, researchers have suggested jointly using OF with alkaline passivators when remedying Cd-polluted soil [16]. Alkaline passivators (such as calcium carbonate, clay minerals, phosphate rock powder, or biochar) can effectively reduce heavy metals’ bioavailability and reduce heavy-metal enrichment in crops [17]. However, previous research has mainly addressed the combined effect on the improvement of the heavy-metal bioavailability and heavy-metal enrichment in crops relative to OF or alkaline passivators alone, comparisons among different joint effects of OF, and different alkaline passivators at different growth stages of crops. Moreover, few studies have focused on food production safety, which has a closed relationship with human health.

Production safety assessment is extremely important, especially when it is hard to test the impact of different remediation methods for Cd-polluted soils. Grey relational analysis (GRA) is an excellent method and is widely used for quantitative description and comparison of the dynamic trend of a system’s development [18]. Erum and Shazia analyzed the relationships among carbon emissions, population, economy, and other factors in Asia through GRA [19]. Soil erosion vulnerability was assessed using the GRA method by Shachi et al. [20]. The GRA method selects the best application combination by calculating the grey correlation degree between various sequences [21,22]. It mainly judges whether they are closely related according to the similarity of a sequence of geometric shapes, reflecting the degree of correlation between multiple factors [23]. GRA is widely applied in many fields (such as economics, industrial technology, and environmental science), with its simple operation, reliable results, and no excessive requirements for sample size [24].

We hypothesized that the Cd toxicity to vegetables could be significantly reduced while increasing the products’ production by jointly applying alkaline passivators with organic fertilizer. To test the hypothesis, three alkaline passivators (i.e., biochar, fish bone, and phosphate rock powder) were separately applied in combination with organic fertilizer to Cd-polluted soil based on pot experiments. This study aimed to (1) study the impact on the Cd toxicity reduction in edible pak choi plants at different growth stages, (2) investigate whether the vegetable production was increased, and (3) assess the food safety using the GRA method.

## 2. Materials and Methods

### 2.1. Experimental Design and Implementation

The experiment was designed with five treatments: (1) without organic fertilizer or passivator (CK), (2) 1% pig manure organic fertilizer (PM), (3) 1% biochar combined with 1% organic fertilizer (CBP), (4) 0.5% phosphate rock powder combined with 1% organic fertilizer (PRP), and (5) 0.5% fish bone meal combined with 1% organic fertilizer (FBP). Each treatment was replicated 9 times, and the total pot number was 45. Three alkaline passivators were selected due to their good performance in heavy-metal stabilization with low costs and abundant resources [25,26].

Each pot was filled with 2 kg of Cd-polluted soil. The soil was sampled from an agricultural field in Liuyang, China. The basic characteristics of the soil, OF, and alkaline passivators are listed in Table 1. The Cd pollution value of the soil was 1.17 mg/kg, which largely exceeded the soil risk screening value of 0.3 mg/kg in the Chinese Soil Environmental Quality Soil Pollution Risk Control Standard for Agricultural Land (GB15618-2018). The soil had a pH of 5.88, a total organic matter content of 21.2 g/kg, and a total cation exchangeable capacity (CEC) content of 8.71 cmol/kg.

In each pot, 8 pak choi seeds were sown, and the variety was Qingnong-8 (*Brassica campestris* L.), which is widely planted in this local region. When the seedlings grew to 2 cm, 3 seedlings with uniform and good growth were kept in each pot. The pots were kept in a greenhouse at 20–25 °C and watered regularly.

### 2.2. Sampling and Determination

Three pots of each treatment were randomly selected to collect plant and soil samples on the 15th, 30th, and 45th days after emergence. The aboveground plant parts were weighed to obtain the fresh edible biomass. Both the aboveground plant and soil samples were dried, ground, and screened. The soil pH was determined using a pH meter. A pH meter (SevenExcellence S400 Basic, METTLER TOLEDO, New York, NY, USA) was used to determine the soil pH value by mixing 20 g of soil with 50 mL of deionized water. The content of available Cd in the soil was extracted with diethyltriamine pentaacetic acid solution (DTPA) and then determined via atomic absorption spectrometry. The plant subsamples were digested in the HNO_3_-HClO_4_ acid mixture (7:1), and soil subsamples were digested in the HNO_3_-HF-H_2_O_2_ acid mixture (5:1:2) to measure the total Cd content via inductively coupled plasma optical emission spectroscopy (ICP-OES, Perkin-Elmer Optima 8 × 00, Perkin-Elmer, MA, USA).

Quality assurance and quality control measures were implemented with nine duplicates of standard reference materials (GBW07401, GBW07603 GSV-2) from the Chinese Academy of Measurement Sciences for each batch of samples. Method blanks were run for background correction and to identify the other sources of the error. Cd standards were detected every 10 samples, with recovery rates within 100 ± 5%. The detection limit (LOD) and the quantification (LOQ) of Cd were 1.86 × 10^−7^ and 1.04 × 10^−6^ mg L^−1^, respectively.

### 2.3. Statistical Analysis

#### 2.3.1. Calculation of Bioenrichment Factor, Absorption Factor, and Transport Factor

The bioenrichment factor (BAF), absorption factor (AF), and transport factor (TF) were calculated and analyzed to indicate the ability of plants to accumulate, uptake, and transport Cd [27,28].

The calculation formulas are as follows:

$$BAF=\frac{PSC}{SC}$$

$$AF=\frac{PUC}{SC}$$

$$TF=\frac{PSC}{PUC}$$

where *PSC* and *PUC* represent the heavy-metal contents in the aboveground and underground parts, respectively, in mg kg^−1^; *SC* represents the available Cd content in the soil, in mg kg^−1^.

#### 2.3.2. Grey Relational Analysis

The soil pH, soil available Cd content, Cd content in the edible parts, BAF, AF, and TF were screened to represent the main factors that affect the safe production of pak choi in Cd-contaminated soil [27,29]. Food safety was assessed using the GRA method, and the brief procedure was conducted as follows:

(1) First, the evaluation index *X* was selected as the soil pH, available Cd content, Cd content in the edible part, BAF, AF, and TF, which are denoted as *X*(*k*), *k* = 1, 2, 3… 6, respectively.
(2) The optimal value of each evaluation index was used to establish the corresponding optimal sequence, which is denoted as *X*_0_(*k*) [23].
(3) The original data were standardized and denoted as *X_i_*(*k*):

When the indexes with a greater value implies a better effect, *X_i_*(*k*) was calculated

$$\mathrm{as}\;X_{i}(k)=\frac{X\left(k\right)-minX_{i}(k)}{maxX_{i}\left(k\right)-minX_{i}(k)};$$

When the indexes with a smaller value indicates a better effect *X_i_*(*k*) was calculated

$$\mathrm{as}\;X_{i}(k)=\frac{maxX_{i}\left(k\right)-X\left(k\right)}{maxX_{i}\left(k\right)-minX_{i}(k)};$$

where *X_i_*(*k*) and *X*(*k*) represent the standardized and the original data, respectively; *minX_i_*(*k*) and *maxX_i_*(*k*) represent the minimum and maximum values of the original data, respectively.

(4) The absolute deviation of grey correlation degree was calculated as:

$$∆min={min}_{i}{min}_{k}\left|X_{i}\left(k\right)-X_{0}(k)\right|\;\mathrm{and}\;∆max={max}_{i}{max}_{k}\left|X_{0}\left(k\right)-X_{i}(k)\right|.$$

(5) The correlation coefficient (*η_i_* (*k*)) between the optimal sequence and the comparison sequence was calculated as:

$$\eta_{i}(k)=\frac{∆min+\rho ∆max}{\left|X_{0}\left(k\right)-X_{i}(k)\right|+\rho ∆max}\;(\mathrm{The}\;\mathrm{resolution}\;\mathrm{coefficient}\;\rho =0.5).$$

(6) Finally, the grey correlation degree (*r*) between the matching application and each index was obtained as:

$$r=\frac{\sum_{i=0}^{n}\eta_{i}(k)}{n}.$$

Descriptive data and statistical analyses were carried out with SPSS v.20.0 and Origin v.2019b, providing the means ± S.D. (standard deviation) of the three replicates. All the plant Cd contents were determined on a dry-weight basis. Statistically significant differences between the different treatments were identified via two-way analysis of variance (ANOVA) using Duncan’s test at a significance level of 0.05. The differences were significant when the significance values were below 0.05.

## 3. Results

### 3.1. Edible Plant Biomass and Cd Content

**Figure 1:**
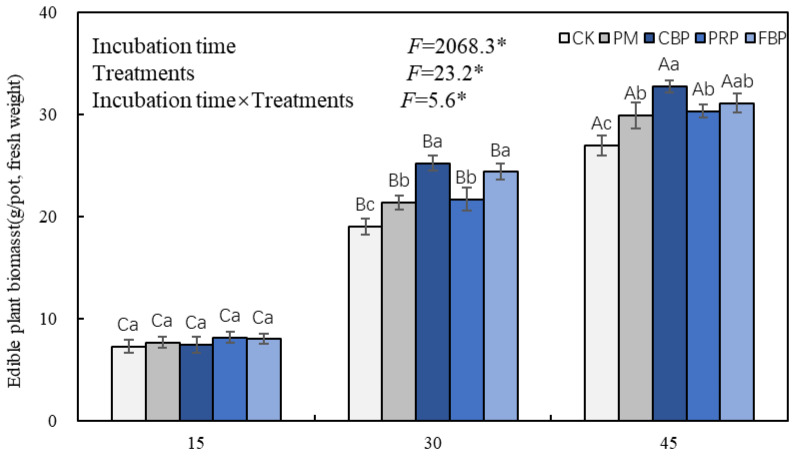
The effect of different treatments on edible fresh biomass. Note: The vertical bars above and within the columns indicate the standard errors (*n* = 3). * implies incubation periods, treatments, and their joint effects induced significant changes in the edible fresh biomass at the 5% level. Different lowercase (uppercase) letters symbolize the significance of the edible fresh biomass across the incubation periods (treatments) at the 5% level according to Duncan’s test. The same notation is presented in the following figures.

As shown in Figure 1, the edible fresh biomass was significantly affected by the treatments, incubation time, and their interaction according to the two-way ANOVA results. The edible fresh biomass significantly increased with the incubation time increasing across all the five treatments. Compared with CK, the treatment-induced promotion of plant growth was non-significant, and no significant difference was observed between the different treatments on the 15th day. The fresh biomass quantity was significantly higher than that of CK on the 30th and on the 45th day: 12.4–32.8% and 10.8–29.6%, respectively, and the CBP treatment performed the best. No significant difference was observed between the treatments of PRP and PM, no matter whether it was the 30th or the 45th day. However, the FBP treatment performed significantly better than the FM or the PRP treatment did on the 30th day.

**Figure 2:**
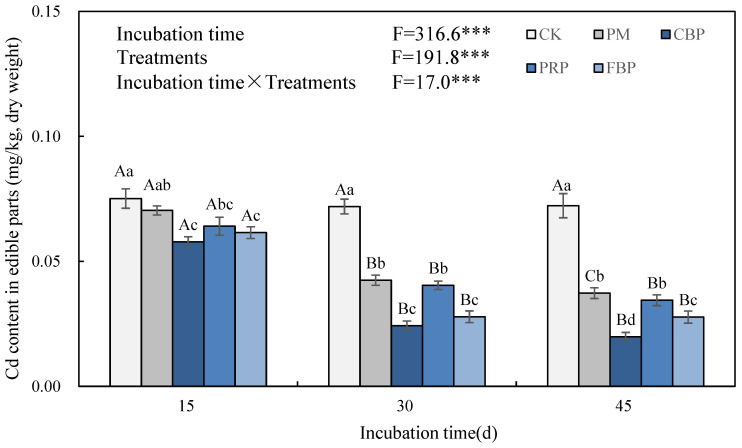
The effect of different treatments on edible plant Cd content. *** implies incubation periods, treatments, and their joint effects induced significant changes in the edible fresh biomass at the 5% level. Different lowercase (uppercase) letters symbolize the significance of the edible fresh biomass across the incubation periods (treatments) at the 5% level according to Duncan’s test. The same notation is presented in the following figures.

Relative to CK, the edible plant Cd contents significantly decreased by 6–48%, 23–72%, 14–52%, and 18–62% in the PM-, CBP-, PRP-, and FBP-treated soils, respectively, except a non-significant increase in the FM-treated soil on the 15th day (Figure 2). The combination treatment of CBP and FBP significantly induced more decrements than FM did across all three incubation periods. However, there was no significant difference between FM and PRP. In particular, the edible plant Cd contents significantly decreased with the incubation time increasing from the 15th day to the 30th day in all the treatments but CK. From the 30th day to the 45th day, the most significant inhibition of plant Cd accumulation was caused by the CBP treatment, although there was a significant interaction between the incubation time and different treatments.

### 3.2. Ability of Plants to Accumulate, Absorb, and Transport Cd

To analyze the ability of plants to accumulate, absorb, and transport Cd, the BAF, AF, and TF values were calculated and are listed in Table 2, respectively. The incubation time induced significant decreases in the BAF and TF values across the four OF treatments, while no significant change was observed in the AF values on the 30th day. Compared with CK, the BAF and TF values in the four treatments significantly decreased, while the AF values significantly increased. However, there was a non-significant difference among the different OF treatments on the 15th day. In particular, no regular phenomenon was observed among the different treatments across the whole incubation period.

### 3.3. Soil pH and Available Cd Content

**Figure 3:**
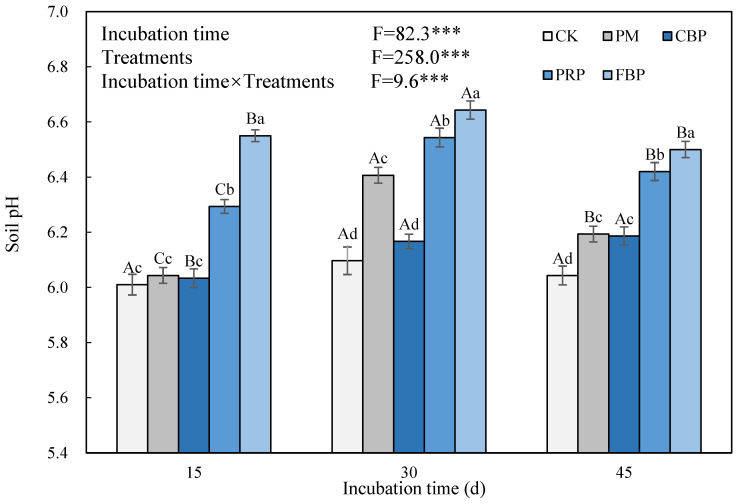
The effect of different treatments on soil pH. *** implies incubation periods, treatments, and their joint effects induced significant changes in the edible fresh biomass at the 5% level. Different lowercase (uppercase) letters symbolize the significance of the edible fresh biomass across the incubation periods (treatments) at the 5% level according to Duncan’s test. The same notation is presented in the following figures.

The incubation time, OF treatments, and their interactions generally had a significant impact on the soil pH (Figure 3). Compared with CK, the PRP and FBP treatments significantly increased soil pH by 0.20–0.57, 0.38–0.57, and 0.37–0.48 units on the 15th, 30th, and 45th days, respectively. The impacts of CBP and PM treatments varied with different incubation periods with non-significant changes on the 15th day. The four OF treatments significantly increased the soil pH after a 30-day incubation period, despite the increments decreasing on the 45th day.

**Figure 4:**
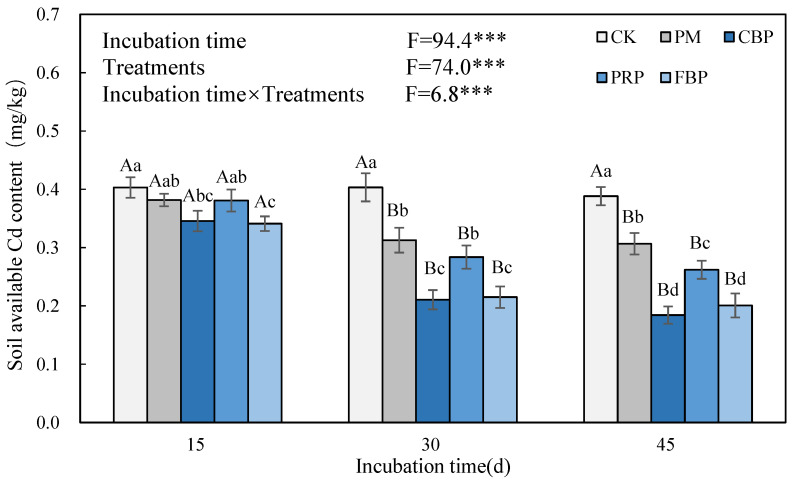
The effect of different treatments on soil available Cd. *** implies incubation periods, treatments, and their joint effects induced significant changes in the edible fresh biomass at the 5% level. Different lowercase (uppercase) letters symbolize the significance of the edible fresh biomass across the incubation periods (treatments) at the 5% level according to Duncan’s test. The same notation is presented in the following figures.

The available Cd contents in the four OF-treated soils significantly decreased both on the 30th and 45th days (Figure 4). On the 15th day, the available Cd content was significantly decreased on average by 14.1% and 15.4% in the CBP- and FBP-treated soil, respectively, while the PM and PRP treatments induced no significant change. The decrements enlarged by 22.3–26.2%, 47.6–52.6%, 29.5–32.5%, and 46.7–48.2% in the PM-, CBP-, PRP-, and FBP-treated soils, respectively, during the 30th and 45th days. Generally, the OF treatments, the incubation time, and their interactions showed significant effects on the available Cd content. According to the Pearson correlation analysis results, the soil pH showed a significant negative relationship with both the soil available Cd and edible plant Cd, which had a significant positive correlation.

### 3.4. Food Safety Assessment via GRA

**Figure 5:**
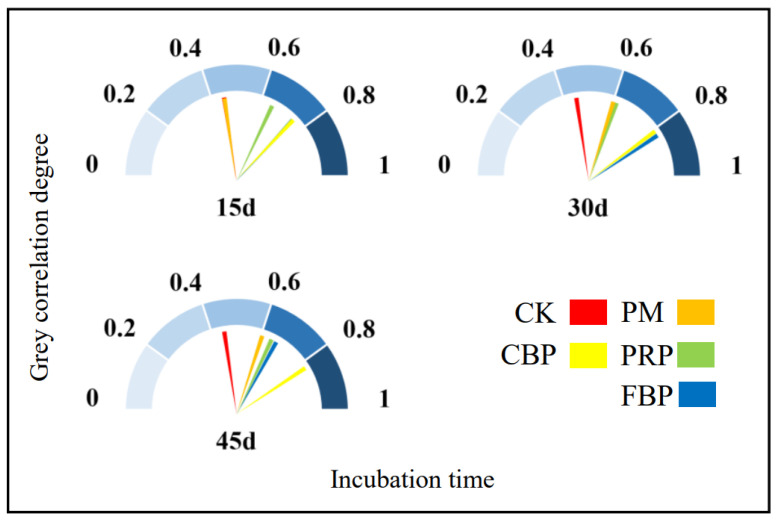
Relationships among soil pH, available Cd, and the effect of different treatments on soil available Cd.

As shown in Figure 5, the r values followed a descending sequence of CBP > FBP > PRP > PM > CK. In detail, the r values of the FBP and CBP treatments were higher than the others on the 15th day, while FBP showed a maximal r value on the 30th day, followed by CBP. However, CBP outperformed FBP and had a maximal r value when the incubation time increased to 45 days. PRP had a higher r value relative to that of CK across all the incubation times, while that of PM was higher solely on the 15th day.

## 4. Discussion

OF is rich in both organic matter and beneficial microorganisms [30,31]. Organic matter highly correlates with soil Cd availability and migration via adsorption, chelation, and complexation [8]. The hydrophilic components of organic matter could bind to Cd^2+^ in soil, change the absorption capacity of soil particles to Cd^2+^, and increase the availability of Cd^2+^ [32]. The high-molecular-weight humic acid released from the decomposition of organic matter strongly binds metals, making them unavailable in soil solutions [33]. Beneficial microorganisms in OF actively reduce plant Cd toxicity by sorbing and enriching Cd or by changing the Cd element content from high to low state [34]. However, previous research indicated that dissolved organic matter released by OF itself and the decomposition process chelates with Cd to improve its plant availability [35]. Low-molecular-weight humic acids released from organic matter decomposition improve metal availability by forming chelates and preventing metal adsorption on solid surfaces [36]. To overcome OF-induced problems in agricultural production, research suggests jointly using OF with alkaline passivators when treating Cd-polluted soil. The addition of a passivator changes the nutrient content of the compost matrix and then changes the microbial community [37], which further affects the passivation of Cd [38].

In this study, we jointly applied alkaline passivators (i.e., biochar, phosphate rock powder, and fish bone meal) and OF to in situ Cd-polluted soil to solve the OF-induced problems in vegetable production. The results showed that the combination of passivators and OF generally performed better than the sole application of OF did in food safety and productivity improvement by reducing the edible plant Cd contents and soil Cd availability while increasing the soil pH. The results are consistent with previous studies, which reported that the combined application of passivators and organic fertilizers increases plant productivity by increasing the soil pH and fertility [39,40].

Agegnehu et al. [41] found that biochar and compost were more effective in improving the soil properties and field crop yields than biochar alone was. The BAF and TF values of the four treatments with the passivator decreased significantly. This indicates that the passivation agent can significantly reduce the enrichment of Cd in the available fraction. At the same time, the transfer of the Cd content from root to shoot was also reduced. In general, BAF and TF values below 1.0 indicate a weak response of shoot to metal concentrations in the soil and a low translocation of Cd from the root to the shoot [42,43]. Among them, adding biochar had the most obvious effect on reducing the quantity of Cd. Sun et al. [44] also showed that biochar is an effective treatment for Cd-contaminated soil. The AF value increased, indicating that the passivator promoted Cd uptake in lettuce roots. The enrichment of Cd in lettuce roots helped to reduce Cd in the soil and did not affect edible parts of the shoot. With the increase in incubation time, the BAF and TF values decreased significantly. Similarly, studies have found that the combined application of a passivator and organic fertilizer reduces the bioavailability of Cd [15], thus further reducing the uptake of cadmium by pak choi.

In this study, the content of Cd in shoot, BAF, and TF significantly decreased after adding passivators. Cd available from soil organisms is transferred to plant roots in the form of organometallic complexes [45,46]. Passivation materials can limit the mobility of heavy metals and reduce their uptake by plants [47]. Reducible and oxidizable Cd represent Cd adsorbed by Fe-Mn oxide and organic matter, respectively [48,49]. These forms do not go directly to the plant roots and are therefore not phytotoxic. However, they are easily affected by changes in the soil pH value, organic matter content, and other factors [50]. Kamran et al. [51] proposed that there is an obvious negative correlation between the pH and bioavailable heavy metals in the soil. Other researchers also suggested that passivators increase soil alkalinity to reduce Cd availability [52,53]. The PRP and PBP treatments caused the most significant increase in the pH value. Passivating material leads to an increase in the soil pH, which may be due to different characteristics in the material, such as basic functional groups (produced by carbonate and single-bond OH groups) and ash content [51,54]. In addition, the soil pH increases after biochar application, which reduces the extractability of heavy metals as new adsorption sites are provided in biochar-treated soils [55]. Changes in the soil pH affect the bioavailability of Cd, which indirectly affects the pak choi yield.

The OF treatment improved soil fertility and promoted the growth of the pak choi yield [56]. The CBP treatment caused the largest decrease in the Cd content in the shoots, while the PM treatment caused the smallest decrease. The content of Cd in the aboveground parts decreased from about 0.07 mg/kg to about 0.03 mg/kg, indicating that organic fertilizer and passivators could significantly inhibit the absorption and transport of Cd in pak choi. However, except for the PM treatment, the other treatments showed no significant decrease between 30 d and 45 d. Feng et al. [57] found that a low concentration of Cd (0–1 mg/kg) could promote the growth of pak choi. When the concentration of Cd is higher than 5 mg/kg, the pak choi yield will be lower. The content of Cd in the aboveground parts decreased gradually. In addition to the reduction of plant availability of Cd due to both the OF and passivators [58], the root growth of pak choi was significantly inhibited under the stress of Cd^2+^. Stem inhibition was not obvious. The results show that Cd was mainly concentrated in the root of pak choi [59]. Most Cd is enriched in the roots of plants, complexed with proteins and polysaccharides to form a precipitation and is fixed, and the part containing Cd migrates aboveground. In addition to root migration, a small part of Cd in the aboveground parts comes from stem and leaf direct absorption [60]. Li et al. [61] also found that pak choi weakly transports Cd and mainly concentrates heavy metals in the roots, which is relatively safe for the human body. These findings suggest that the combination of a passivator and OF can increase the aboveground biomass and reduce the absorption of Cd in pak choi, which is of great significance for the safe production of pak choi.

Biochar has a porous structure with many oxygen-containing functional groups on the surface, which can effectively adsorb Cu, Pb, Zn, and Cd [62]. It can significantly reduce the bioavailability of heavy metals. Phosphate rock powder is a phosphate fertilizer, which can be passivated via ion exchange adsorption, precipitation, and the dissolution of Pb, Zn, and Cd [63]. The main components of bone meal are mineral salt, protein, and fat. Fish bone meal can effectively raise the soil pH value and reduce the biological activity level of Cd [25]. This study showed that the combined application of passivators and OF directly affects the content of available Cd in soil. Compared with CK, the weight of the edible part of pak choi was significantly increased with each combination treatment. Among them, the CBP and FBP treatments had the best effect. The difference between them was not significant, which could increase the aboveground weight of pak choi by 15.3–32.8%. These treatments could indirectly affect the available Cd content by affecting the soil pH. It was found that the pH value of soil with the organic fertilizer and passivating agent was higher than that treated with CK, and the content of available Cd was lower than that in the soil treated with CK. The soil pH values after the PRP and FBP treatments were significantly higher than those subjected to the other treatments, with an increase of 0.28–0.54 units. The FBP treatment had the best effect (Figure 1). Compared with the CK treatment, FBP reduced the available Cd content in the soil by 46.7–48.2% (Figure 2). The soil pH value was negatively correlated with the available Cd content in the soil. The higher the pH value was, the lower the available Cd content was. Soil pH determines the form of heavy metals in soil to a certain extent. Increasing the pH will cause some of the ion exchange parts of Cd, Pb, and Zn to precipitate hydroxide, thus reducing the available content in the soil [64]. The application of a pig manure organic fertilizer could significantly reduce the content of available Cd in the soil by 0–13.33% and thus inhibit the absorption and accumulation of Cd by crops [6]. The reason for this may be that an organic fertilizer can improve the pH value of soil and convert some heavy-metal ions into sediment, thus reducing the content of available Cd [26]. It can be seen from Figure 2 that the combined application of fish bone meal and an organic fertilizer can significantly reduce the content of available Cd in soils. It is speculated that the content of available Cd in acidic soil decreases by 2.9–17% after the application of fish bone meal. This is because the main component of fish bone meal is alkaline mineral salt, which can directly improve the pH value of soil when it is applied to soil. Moreover, it can react with heavy-metal ions on the surface, thus reducing the activity of heavy metals [65]. The amount of negative charge carried by soil colloids will increase with the increase in the pH, enhancing the adsorption affinity of soil for heavy-metal ions and playing a role in fixing heavy-metal ions [66].

The application of an organic fertilizer alone or the combined application of passivators and an organic fertilizer could reduce the content of available Cd in the soil and reduce the biological activity and harm caused by Cd. An organic fertilizer can promote the secretion of plant roots and microorganisms and activate heavy metals [67]. Hu et al. [16] also found that the combined application of organic fertilizer and passivators could significantly reduce the content of available Cd in soils. Biochar has a good structure and large specific surface area and is porous, and it is beneficial for the adsorption, complexation, and ion exchange of Cd and other heavy metals [68]. This can help reduce Cd damage to crops. These findings suggest that the combined application of passivators and an organic fertilizer could directly affect the content of available Cd in soil or indirectly affect the pH value of soil, thus reducing the uptake of Cd by crops.

## 5. Conclusions

In this study, the hypothesis that the toxicity of Cd to vegetables can be significantly reduced by the combined application of an alkaline passivator and organic fertilizer, while increasing product yield, was verified via a pot experiment. The results showed that the addition of organic fertilizers and passivators resulted in an increase in the soil pH while decreasing the Cd content in the edible parts and the available Cd content in the soil and increasing the biomass. The combined application of an organic fertilizer and passivator resulted in the best reduction of the cadmium content in the edible fraction and soil available cadmium content by 52.3–72.6% and 32.5–52.6%, respectively. The combined application of biochar and an organic fertilizer showed the best performance in reducing the Cd content in the edible portions and available Cd content in the soil. These results are supported by the highest GRA value of the treatment. Compared with the control, the migration coefficients of Cd were reduced by 61.9% and 50.9–55.0% via the application of organic fertilizer alone and the combined application of a passivator, respectively, which effectively blocked the migration of Cd between the plants and soil. These results suggest that the combined application of biochar and an organic fertilizer perform best in reducing the risk of potential Cd contamination, thus emphasizing vegetable food safety. This study provides a potential green technology for toxicity reduction and food safety in Cd-polluted soil, with a low cost and a high efficiency.

## Figures and Tables

**Table 1:** Basic characteristics of soil, organic fertilizer, and passivators.

Materials	Basic Characteristics			
	pH	Total Cd (mg kg^−1^)	CEC (cmol kg^−1^)	Organic Matter (g kg^−1^)
Soil	5.88	1.17	8.71	21.2
Organic fertilizer	8.04	0.46	-	485
Biochar	9.37	0.47	118.64	129.6
Phosphate rock powder	8.42	0.07	-	4.4
Fish bone meal	7.91	0.66	8.05	345

**Table 2:** Bioenrichment factor (BAF), absorption factor (AF), and transport factor (TF) of plant Cd subjected to different treatments.

Treatments	BAF			AF			TF		
	15 d	30 d	45 d	15 d	30 d	45 d	15 d	30 d	45 d
CK	0.186 ^Aa^	0.179 ^Aa^	0.186 ^Aa^	0.332 ^Ab^	0.345 ^Ad^	0.331 ^Ac^	0.563 ^Aa^	0.521 ^Aa^	0.564 ^Aa^
PM	0.184 ^Aa^	0.136 ^Bb^	0.122 ^Bbc^	0.455 ^Ba^	0.632 ^Aa^	0.567 ^Aa^	0.406 ^Ab^	0.217 ^Bb^	0.215 ^Bc^
CBP	0.168 ^Aa^	0.116 ^Bb^	0.109 ^Bc^	0.404 ^Aa^	0.455 ^Ac^	0.391 ^Ab^	0.417 ^Ab^	0.254 ^Bb^	0.277 ^Bb^
PRP	0.169 ^Aa^	0.144 ^Ab^	0.132 ^Bbc^	0.445 ^Ba^	0.534 ^Ab^	0.519 ^Aa^	0.381 ^Ab^	0.268 ^Bb^	0.254 ^Bbc^
FBP	0.181 ^Aa^	0.130 ^Bb^	0.139 ^Bb^	0.467 ^Ba^	0.562 ^Aab^	0.518 ^ABa^	0.387 ^Ab^	0.232 ^Bb^	0.269 ^Bb^

Note: Different lowercase (uppercase) letters symbolize significance in indexes across the incubation periods (treatments) at the 5% level according to Duncan’s test.

## Data Availability

Data will be made available on request.
